# Plant extracellular vesicles: Trojan horses of cross‐kingdom warfare

**DOI:** 10.1096/fba.2021-00040

**Published:** 2021-06-27

**Authors:** Baoye He, Rachael Hamby, Hailing Jin

**Affiliations:** ^1^ Department of Microbiology and Plant Pathology Center for Plant Cell Biology Institute for Integrative Genome Biology University of California Riverside CA USA

**Keywords:** cell‐to‐cell communication, cross‐kingdom RNAi, exosome, extracellular vesicles, plant immunity, small RNA

## Abstract

Plants communicate with their interacting microorganisms through the exchange of functional molecules. This communication is critical for plant immunity, for pathogen virulence, and for establishing and maintaining symbioses. Extracellular vesicles (EVs) are lipid bilayer‐enclosed spheres that are released by both the host and the microbe into the extracellular environment. Emerging evidence has shown that EVs play a prominent role in plant–microbe interactions by safely transporting functional molecules, such as proteins and RNAs to interacting organisms. Recent studies revealed that plant EVs deliver fungal gene‐targeting small RNAs into fungal pathogens to suppress infection via cross‐kingdom RNA interference (RNAi). In this review, we focus on the recent advances in our understanding of plant EVs and their role in plant–microbe interactions.

## 1 INTRODUCTION

Communication between cells, whether within one organism or between interacting organisms, is critical for the function of multicellular organisms and for host–microbe interactions. Functional molecules, including proteins, nucleic acids, lipids, and metabolites, particularly regulatory small RNAs (sRNAs), move between hosts and interacting microorganisms to influence a series of physiological and pathological processes.^1^, ^2^ Extracellular vesicles (EVs) have emerged as a major pathway to achieve precise and efficient transport of functional molecules between cells and organisms,^2^, ^3^ specifically by protecting their cargoes from degradation in the process of delivery.^4^ EVs are secreted by eukaryotic parasites as well as their animal hosts and can manipulate cellular processes in both partners.^5^ In plant–microbe interactions, plants utilize EVs to deliver sRNAs into fungal pathogens and suppress the expression of fungal virulence‐related genes.^6^ The biogenesis and function of EVs in animal cells and animal–pathogen communication have been well studied, but our knowledge of EVs in plant systems remains rudimentary. Here, we will review the recent discoveries in plant EV biogenesis, the role of plant EVs in plant–microbe interactions, the mechanisms of EV sRNA loading, and the potential applications of these discoveries for preventing plant disease.

## 2 HETEROGENEITY OF PLANT EVs

**FIGURE 1:**
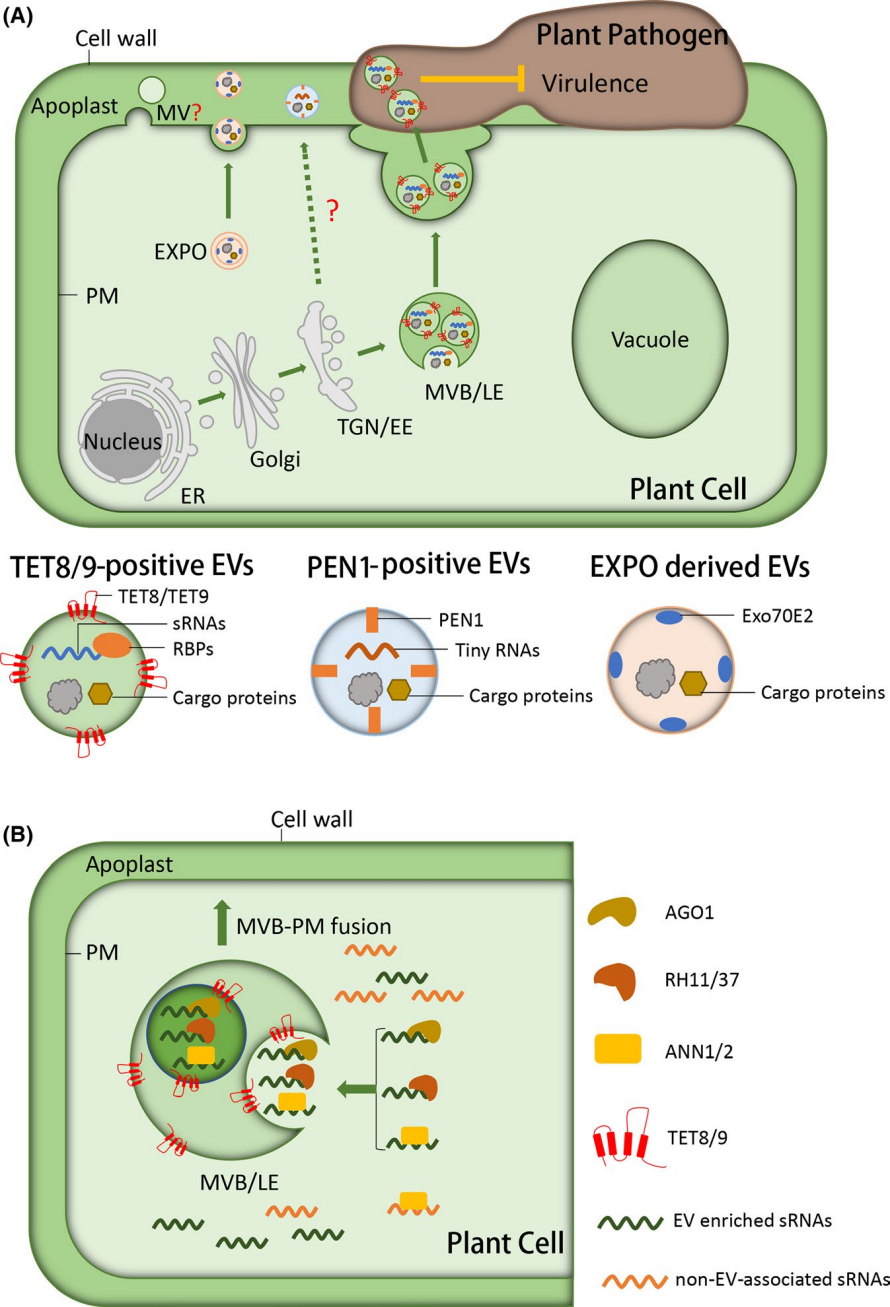
Plant EVs communicate with interacting microbes. A, A heterogeneous population of EVs exists in plants. After fusion with PM, MVBs release TET8/9‐positive EVs into the extracellular space. TET8/9‐positive EVs contain defense‐related molecules such as host‐derived sRNAs, defense proteins, and RBPs that function in sRNA sorting and stabilization in EVs. These host‐derived defense molecules can be internalized by plant pathogens to suppress pathogen virulence. Another class of EVs is PEN1‐positive EVs, which contains tiny RNAs. But the biogenesis pathway of PEN1‐positive EVs and whether they contribute to plant immune responses or cross‐kingdom regulation is not yet clear. EXPO produces another class of single‐membrane‐bound EVs, which may aide in the re‐localization of defense‐related molecules during pathogen invasion. MVs may also be secreted by plant cells through outward budding directly from the PM. The three major classes of plant EVs are presented in the bottom panel individually. The question mark indicates a prediction that has not yet been validated experimentally. B, RNA‐binding proteins contribute to sRNA loading into plant extracellular vesicles. Arabidopsis Argonaute 1 (AGO1), RNA helicase (RH) 11, and RH37 selectively bind to EV‐enriched sRNAs but not to non‐EV‐associated sRNAs, annexin (ANN) 1 and ANN2 bind to sRNAs non‐specifically. These RNA‐binding proteins with EV‐enriched sRNAs are targeted for capture into a bud invaginating into the MVB. ER, endoplasmic reticulum; EV, extracellular vesicle; EXPO, exocyst‐positive organelle; LE, late endosome; MV, microvesicle; MVB, multivesicular body; PEN1, Penetration 1; PM, plasma membrane; RBPs, RNA‐binding proteins; TET, Tetraspanin; TGN/EE, trans‐Golgi network/early endosome

In mammalian systems, EVs are divided into multiple classes based on their distinct biogenesis pathways and specific protein markers, including exosomes, microvesicles, and apoptotic bodies.^7^ Exosomes originate from multivesicular bodies (MVBs), which then fuse with the plasma membrane, to release their intraluminal vesicles (ILVs) into the extracellular space, forming exosomes.^3^ Restricted by the size of ILVs, the diameter of exosomes ranges from 30 to 100 nm.^7^ Microvesicles originate from the direct outward budding of the plasma membrane. The size of microvesicles ranges from 50 to 1000 nm but can be even larger in some cancer cells.^8^ An additional class, apoptotic bodies, arise from blebbing of the apoptotic cell membrane and can be over 1 μm in diameter.^9^ In plants, EVs were first observed in the 1960s in chemically fixed carrot cells using electron microscopy. In these initial observations, different sizes of EVs were detected.^10^, ^11^ Now, EVs have been discovered in extracellular fluids of leaves, roots, fruits, and imbibing seeds.^6^, ^12^, ^13^, ^14^ In recent years, an increasing number of studies have implied that, like animal cells, there exists a heterogeneous population of EVs in plants (Figure 1A).

In mammalian systems, tetraspanin proteins, such as CD9, CD63, CD37, CD81, or CD82, are enriched in the membranes of exosomes and often used as exosome biomarkers.^15^ The model plant, *Arabidopsis thaliana*, has 17 TETRASPANIN (TET)‐like genes. Despite their limited amino acid sequence similarity with animal tetraspanin proteins, they share conserved structural hallmarks, including four transmembrane domains (TM1–TM4), a small extracellular loop (ECL1), an intracellular loop (ICL), and a large extracellular loop (ECL2).^16^ Two of the *Arabidopsis* TETs, TET8 and TET9, are specifically induced upon infection by fungal pathogen *Botrytis cinerea*.^6^ Meanwhile, both co‐localize with the *Arabidopsis* MVB‐marker Rab5‐like GTPase ARA6 inside the cell, and also co‐localize in EVs which are enriched at fungal infection sites.^6^ These TET8‐positive EVs were highly enriched in the fraction collected at ultracentrifugation speeds of 100,000 *g* from leaf apoplastic fluid.^6^ In density gradient ultracentrifugation, TET8‐positive EVs are enriched in the fractions at the density of 1.12–1.19 g/ml, which is consistent with the density of exosomes in animal systems.^17^, ^18^ Meanwhile, the plant EV‐enriched sRNAs and cargo proteins are also present in the same fraction as TET8.^18^ These results suggest that TET8‐positive EVs can be considered *bona fide* plant exosomes, and transport sRNAs. Tetraspanins play a crucial role in EV formation and function in mammalian cells.^15^ In plants, the *tet8* single mutant or the *tet8 tet9* double mutant plants displayed decreased secretion of EVs and sRNAs, and enhanced susceptibility to *B. cinerea* infection.^6^, ^18^ Further investigation revealed that the amount of an EV‐enriched lipid, glycosylinositolphosphoceramides (GIPCs), was dramatically decreased by over fourfold in *tet8* total leaf extracts.^19^ These results indicate that TET8 mediates the production of EVs in association with GIPCs.

PENETRATION 1(PEN1)‐positive EVs represent another class of plant EVs. PEN1 is a plasma membrane‐associated plant‐specific syntaxin.^20^ It was first identified by mutational analysis in *Arabidopsis* to screen mutants that were disabled in non‐host penetration resistance against barley powdery mildew, *Blumeria graminis f. sp. Hordei*.^21^ The secretion of PEN1 depends on an ADP ribosylation factor‐GTP exchange factor (ARF‐GEF), GNOM, which mediates recycling endosome trafficking rather than MVB pathway.^22^ PEN1‐positive EVs are enriched at a lower ultracentrifugation speed (40,000 *g*) than TET8‐EVs from *Arabidopsis* leaf apoplastic fluid.^14^ Furthermore, PEN1 does not co‐localize with MVB marker ARA6 in plant cells and the PEN1‐positive EVs are enriched in the gradient fraction of 1.029–1.056 g/ml.^14^ Additionally, when TET8‐GFP and mCherry‐PEN1 were co‐expressed in *Arabidopsis*, distinct GFP‐labeled and mCherry‐labeled EVs were observed in isolated EVs.^18^ These results indicate that TET8‐ and PEN1‐positive EVs represent distinct classes of plant EVs, and likely possess different biogenesis pathways (Figure 1A).

EXPO, an exocyst‐positive organelle is another source of plant EVs (Figure 1A). It is associated with Exo70E2, which is an homolog of the yeast and animal exocyst protein Exo70, in *Arabidopsis* and tobacco (*Nicotiana tabacum*) suspension cells.^23^ EXPO does not co‐localize with any known organelle markers, including markers of the Golgi apparatus, the trans‐Golgi network/early endosome, or MVBs in plants.^23^ Immunogold labeling of sections cut from high‐pressure frozen samples of wild‐type *Arabidopsis* cells and transgenic BY‐2 cells expressing Exo70E2‐GFP reveals that EXPOs are spherical double‐membrane structures. After fusion with the plasma membrane, EXPO releases single‐membrane‐bound vesicles to the extracellular space.^23^ Several arabinogalactan glycosyltransferases involved in arabinogalactan O‐glycosylation have been found in the EXPO, indicating that GATLs could be co‐secreted to the apoplast via the EXPO.^24^ The mechanism of EXPO biogenesis, and whether the cargoes of EXPO are functional is still unclear.

EVs are also present in fruits, such as grapes and coconuts.^25^, ^26^ Exosome‐like nanoparticles have been isolated from grape juice using differential centrifugation and sucrose gradient methods. The size of grape EVs is between 50 and 300 nm in diameter, similar to the size of exosomes. They may function in the activation of intestinal stem cell proliferation and remodeling of intestinal stem cells in response to pathological triggers.^25^ Additionally, exosome‐like nanoparticles were observed and detected in coconut water by scanning electron microscopy (SEM), fluorescence microscopy, and dynamic light scattering (DLS), although their biological function is unclear.^26^ Olive (*Olea europaea*) pollen grains release nanovesicles during in vitro pollen germination and pollen tube growth, named pollensomes.^27^ Electron microscopy analysis has revealed that these pollensomes represent a heterogeneous population of round‐shaped nanovesicles. Pollensomes size ranges from 28 to 60 nm in diameter with densities ranging from 1.24 to 1.29 g/ml in a sucrose gradient. Pollensomes may provide an alternative way of protein secretion during the processes of pollen germination and pollen tube growth, which are key steps for successful fertilization in plants.^27^

## 3 METHODS OF EV ISOLATION IN PLANTS

In order to elucidate the various functions and distinct subclasses of plant EVs, it is critical to develop efficient and effective EV isolation protocols for use in plant systems. Methods for plant EV isolation are based generally on established mammalian EV separation protocols. Differential ultracentrifugation is the most conventional EV isolation method.^28^ In this method, large and medium vesicles and membrane structures are eliminated by successive centrifugations (2000 *g* and 10,000 *g*) at increasing speeds, which prevents the artificial creation of small vesicles from large ones by direct high‐speed centrifugation.^7^ Small vesicles are then sedimented by ultracentrifugation at 100,000 *g*.^7^ However, this final ultracentrifugation step only allows for the enrichment of small‐sized EVs and cannot distinguish between different subclasses of EVs or protein aggregates of similar size. A more specific method, density gradient ultracentrifugation, enables further separation of membrane‐enclosed vesicles from aggregates of proteins, and the separation of similarly sized EVs with different densities.^29^ In plants, two gradients, sucrose and iodixanol, have been used to further separate different EVs.^14^, ^18^

Immunoaffinity isolation is the most precise method to isolate specific classes of EVs. It takes advantage of EV surface protein markers such as tetraspanin proteins, CD63, CD9, and CD81.^30^, ^31^, ^32^, ^33^ In this method, EV samples isolated by ultracentrifugation are incubated with beads coated with antibodies for EV surface proteins. After washing the beads, only the antibody‐specific binding EVs can be isolated.^30^, ^32^ This method can further prevent cytoplasmic protein or RNA from contaminating isolated EVs. In plants, a native antibody that can specifically recognize the ECL2 domain of TET8 has been generated to specifically isolate TET8‐positive EVs.^18^ The EV‐localized sRNA and protein cargos are clearly detectable in immunoaffinity purified TET8‐positive EVs.^18^ To remove the contaminating RNA and protein molecules that non‐specifically attach to the EV surface or co‐sediment with EVs, nuclease and protease treatments of EVs are widely performed.^29^ Both sRNA and protein cargos contained within EVs are protected from nuclease and protease digestion, unless Triton X‐100 is added to rupture the EVs, demonstrating that plant EVs can indeed protect nucleic acid and protein cargos for transportation.^6^, ^18^ This work has established the initial framework for researching plant EVs.

## 4 PROTEIN TRANSPORT FUNCTION OF EVs IN PLANT–MICROBE INTERACTIONS

As safe vehicles to deliver functional and regulatory components (such as nucleic acids, lipids, and proteins) to other cells or interacting organisms, EVs play prominent roles in communication between interacting organisms. In animal systems, several parasites have been shown to release EVs into host cells to manipulate host immune responses.^5^, ^34^, ^35^, ^36^ *Leishmania*, the causative agent of tropical and sub‐tropical infections termed the leishmaniases, can release exosomes into macrophages.^35^ The incubation of macrophages with *Leishmania* exosomes selectively induced secretion of interleukin‐8, which may facilitate the pathogen infection.^35^ The gastrointestinal nematode, or helminth, *Heligmosomoides polygyrus* utilizes exosomes to deliver miRNAs into mouse host cells to suppress inflammation and innate immune responses during infection.^36^

Emerging evidence also indicates that plant EVs are critical for communication with their interacting microbes. Specifically, EVs are important for antimicrobial defense. Infection with fungal pathogen *B. cinerea* or bacterial pathogen *Pseudomonas syringae pv* tomato DC3000 stimulates the secretion of plant EVs, which indicate the important role of EVs in plant–pathogen interactions.^6^, ^14^ Recently, protein cargos involved in antimicrobial defense, including the glucosinolate transporters PEN3 and NRT1 as well as the myrosinase EPITHIOSPECIFIER MODIFIER1,^26^ have been identified inside *Arabidopsis* EVs.^14^ PEN3 is involved in immunity against powdery mildew fungus, *Golovinomyces orontii* and *P. syringae pv* tomato DC3000 bacteria, and the plant glucosinolate‐myrosinase defensive system is activated only under tissue damage caused by pathogens, insects, or other herbivores.^37^, ^38^, ^39^ This suggests that plant EVs may function as concentrated packets of antimicrobial molecules and compounds. The plant EV proteome was also enriched in various immunity‐related membrane trafficking proteins, such as PEN1 (Syntaxin‐121), Syntaxin‐122, and Syntaxin‐132, further supporting the conclusion that plant EVs are also involved in protein transport during immune signaling.^14^

## 5 RNA‐BINDING PROTEINS CONTRIBUTE TO SELECTIVE LOADING AND STABILIZATION OF sRNAs IN EVs

Based on plant EV sRNA profiling analysis, a specific group of plant sRNAs were detected in EVs,^6^ which suggests that a regulatory process for selective loading of sRNAs into EVs exists in plants. Using mass spectrometry (MS) analysis, He et al. identified a group of RNA‐binding proteins (RBPs), including Argonaute protein 1 (AGO1), DEAD‐box ATP‐dependent RNA helicases 11 (RH11), RH37, RH52, Annexin1 (ANN1), and ANN2 in *Arabidopsis* EVs that isolated at 100,000 *g*.^18^ These RBPs co‐localize with TET8‐positive EVs, and could be detected by Western blot analysis in these TET8‐positive exosomes even after trypsin digestion.^18^ Among these RBPs, AGO1 and RNA helicase proteins can specifically bind EV‐enriched sRNAs in both total RNA extracts and EV fraction. In immunocapture‐purified TET8‐positive exosomes, only the AGO1‐, RH11‐, and RH37‐bound sRNAs, but not AGO2‐ or AGO4‐bound sRNAs were detected, suggesting that AGO1 and RH11/37 contribute to selective sRNA loading into exosomes (Figure 1B). Annexins bind to sRNAs non‐specifically and are not involved in the selective loading process. Moreover, the sRNA levels are reduced in EVs isolated from the *ago1* mutant, and the double mutants of both *rh11rh37* and *ann1ann2*, suggesting that all of these RBPs stabilize the sRNAs in EVs. Furthermore, *rh11rh37* and *ann1ann2* mutants are more susceptible to *B. cinerea* in comparison to wild‐type plants. The expression of fungal virulence‐related genes that are targeted by plant secreted sRNAs were de‐repressed in *B. cinerea* that were collected from *rh11rh37* and *ann1ann2* mutants.^18^ These results how that EV‐associated RBPs contribute to plant immunity by selective loading and stabilization of sRNAs in plant EVs.

EVs are also involved in arbuscular mycorrhizal symbioses and have been observed in the interface of plants and symbiotic arbuscular mycorrhizal fungus.^13^ During the formation and maturation of the arbuscular mycorrhiza, plant MVBs have been observed fusing with the host‐derived peri‐arbuscular membrane (PAM) in areas where plant and fungi interact.^13^ Whether these EVs contain RNAs cargos, especially sRNAs, remains to be established.

## 6 CROSS‐KINGDOM RNAi

Of the many emerging roles of EVs in plant systems, perhaps the most intriguing one is their critical role in cross‐kingdom RNA interference (RNAi). Cross‐kingdom RNAi is the transport of sRNAs between interacting organisms, which target and silence genes in the counter party. This communication mechanism was first discovered in the fungal pathogen, *B*. *cinerea*, which can deliver sRNAs into multiple plant hosts, including *Arabidopsis* and tomato. Once inside plant cells, these fungal sRNAs hijack the plant RNAi machinery protein, AGO1 to silence host immune response genes.^40^ Soon after this initial discovery, cross‐kingdom RNAi was demonstrated to be bidirectional, plants also send sRNAs into *B. cinerea* in order to target and silence key fungal virulence‐related genes.^6^, ^41^, ^42^

Since the discovery and characterization of cross‐kingdom RNAi, this phenomenon has been observed in a variety of interacting organisms. In addition to fungal pathogens, oomycete pathogen *Hyaloperonospora arabidopsidis* also transport sRNAs into their plant hosts and utilize host AGO1 to silence plant genes.^43^ The parasitic plant, *Cuscuta* *campestris*, transports miRNAs into host plants to silence defense response genes.^44^ Cross‐kingdom RNAi can also function in symbiotic interactions. Plant bacterial symbiont, *Rhizobium*, although has no conventional RNAi machinery, can generate sRNA‐like RNAs from transfer RNA (tRNA) degradation. These tRNA‐derived sRNAs are delivered into soybean cells and are loaded into soybean AGO1 to silence soybean genes, which helps to establish the plant–bacteria symbiosis.^45^ Outside of plant interaction systems, cross‐kingdom RNAi has been observed in animal–pathogen or parasite interactions. For example, the gastrointestinal nematode *H*. *polygyrus* sends sRNAs into mammalian gut cells in order to target and silence immunity and inflammation‐related genes.^36^ The fungal pathogen of mosquito, *Beauveria bassiana* transfers a miRNA to the host cells and hijacks mosquito AGO1 to silence host immunity gene *Toll receptor ligand Spätzle 4*.^46^

In most cases, the precise mechanisms underlying cross‐kingdom RNAi transport remain unclear. However, discoveries in plant–fungal and mammal–parasite interactions both suggest the EVs are a major mechanism of interspecies RNA transport.^6^, ^36^ In 2014, Amy Buck's research group discovered that *H. polygyrus* packages sRNAs into EVs, specifically exosomes, to deliver sRNAs into mouse intestinal epithelial cells.^36^ Following this initial finding, a growing number of papers indicates that other parasites utilize the same strategy for sRNA delivery.^47^, ^48^ In plant systems, Cai et al. discovered that the host plant *Arabidopsis* packages fungal gene‐targeting sRNAs in TET8‐positive EVs for delivery into the pathogen *B. cinerea*.^6^ Specifically, Cai et al, found a specific set of plant sRNAs localized in the TET8‐associated EVs.^6^ To confirm the EV localization of these sRNAs, a series of verification including nuclease treatment of purified EVs, high‐speed density gradient ultracentrifugation, and EV immunoaffinity isolation with TET8 antibody were performed.^18^ This work clearly demonstrates that sRNAs are located within TET8‐positive EVs. Furthermore, these TET8‐positive EVs can be efficiently taken up by *B. cinerea* fungal cells.^6^ After taken up by fungal cells, EV delivered sRNAs are released to suppress critical fungal target genes, including vacuolar protein sorting 51 (*Vps51*), a large subunit of the dynactin complex (*DCTN1*) and a suppressor of actin‐like phosphoinositide phosphatase (*SAC1*), which coordinates vesicle trafficking and plays important roles in *B. cinerea* pathogenicity.^27^ In PEN1‐labeled EVs, a group of “tiny RNAs,” which are 10–17 nucleotides in length and derived mainly from the positive strand of mRNA transcripts, have also been found. However, the biological function of these tiny RNAs is still unclear.^49^ A similar phenomenon has been discovered between plants and oomycete pathogen *Phytophthora capsici*.^50^ Under infection by *P. capsici*, *Arabidopsis* delivers secondary phasiRNAs from PPR gene clusters into the pathogen, likely using EVs, to silence target genes in the *P. capsici*.^50^

Intriguingly, ingested plant EVs can shape the mammalian gut microbiome through cross‐kingdom RNAi. Specifically, miRNAs encapsulated in ginger EVs can be taken up by gut microbiota after ingestion, where they target and silence microbe genes, influencing microbiome community composition.^51^ However, bacteria do not have conventional RNAi machinery, it is still not clear how host sRNAs manipulate the expression of bacterial genes. Recent discovery of plant AGO1 protein being secreted together with the sRNAs in EVs led us to hypothesize that host AGO proteins may transport and function with associated host sRNAs in silencing bacterial genes. Taken together, these studies suggest that in plants, as well as animals, EVs are a major mechanism of sRNA transport.

## 7 RNA‐BASED TRANSLATIONAL APPLICATIONS

The critical role of EVs in cross‐kingdom RNAi can be leveraged into innovative plant protection strategies. In one strategy, host‐induced gene silencing (HIGS), plants are genetically engineered to express pathogen/pest gene‐targeting double‐stranded RNAs, which are processed into sRNAs. Subsequently, these sRNAs are transported into the pest/pathogen where they silence key virulence‐related genes to suppress infection.^52^, ^53^ This strategy has been successfully utilized to control both fungal pathogens and insect pests in plants. However, a key drawback with HIGS approaches is that they rely on the generation of transgenic plants, which is still technically challenging in many crop species. Additionally, the cost of overcoming regulatory hurdles necessary for bringing a GMO product to the market further limits the feasibility of the HIGS approach.

An alternative to HIGS, spray‐induced gene silencing (SIGS), requires the direct application of pathogen gene‐targeting RNAs onto plant material, circumventing the need for genetic engineering. Recent discovery of fungal RNA uptake makes SIGS possible to control fungal diseases in crops.^41^, ^54^, ^55^ SIGS approaches have been successfully used to prevent fungal infections in both monocot and dicot plants, as well as in postharvest materials.^41^, ^54^, ^56^ Because RNA is already present in most food, these RNAs are likely safe for human consumption. Furthermore, SIGS is an eco‐friendly alternative to traditional fungicides, as RNAs degrade within 2 days of soil application.^57^ Unfortunately, this rapid degradation is a major hurdle that must be overcome before widespread SIGS applications. One strategy for enhancing RNA stability is to package them within nanoparticles, such as clay nanosheets, which can stabilize RNAs on plant tissue for up to 30 days.^58^ In clinical contexts, lipid nanoparticles, which complex with RNAs to form liposomes, can package and stabilize therapeutic RNA treatments in the bloodstream.^59^ The recently developed mRNA vaccine of severe acute respiratory syndrome coronavirus 2 (SARS‐CoV‐2), the virus that causes COVID‐19, is also encapsulated in lipid nanoparticles to facilitate the delivery into human cells.^60^, ^61^ This strategy may work particularly well in plant–fungal systems, as the liposome encapsulated RNAs mimics the natural delivery system of plant sRNAs in EVs to fungal pathogens.

## 8 CONCLUSION

Though decades of research have been performed on EVs in animal systems, plant researchers are just beginning to scratch the surface of the multitude of complex roles EVs have in plant systems. Though first observed in the 1960s, plant EVs have garnered little research attention until recently. Indeed, reports of EVs in organisms with thick cell walls have previously been largely overlooked because a mechanism for EVs to cross cell walls was unknown. Recent studies in fungi however, clearly indicate that cell walls are viscoelastic and dynamic in nature, and can stretch and accommodate the passage of large molecules, including EVs.^62^

Current methods for EV isolation in plants have largely been adapted from existing EV isolation protocols developed in animals. Using these isolation methods, it has been discovered, that, similar to animals, plants possess a variety of EV subclasses derived from different biogenesis pathways. These EVs are critical for the transport of proteins and small RNAs from plants to their microbe partners and pathogens. Further research into the contents of EVs and their roles in specific plant–pathogen communication mechanisms, such as cross‐kingdom RNAi, will play a crucial role in the development of novel plant protection techniques. Beyond the obvious agricultural applications of plant EV research, increasing evidence indicates that plant EVs can be used in medical applications. As it has already been demonstrated, dietary EVs can impact microbiome composition.^51^ This suggests that it may be possible to package therapeutic sRNAs or medications in plant EVs for transport to target cells in animal systems.

The field of plant EVs is still in its infancy. Early advances have indicated the crucial role of EVs in plant–microbe interactions, especially in cross‐kingdom RNAi. We expect new breakthroughs as this field of research matures. Beyond the basic goal of better understanding both plant physiology and plant–microbe interactions, delving deeper into the world of plant EVs can provide novel solutions to problems in both the agricultural and medical sectors, through innovative crop protection strategies and therapeutic delivery systems.
